# Sudden and Unexpected Death in Children

**Published:** 1904-12-24

**Authors:** 


### SUDDEN AND UNEXPECTED DEATH IN CHILDREN.

At a meeting of the Society for the Study of Disease in Children the above subject was discussed in all its bearings. The medical side of the question was introduced by Dr. C. J. Macalister, Dr. J. Porter Parkinson, and Mr. J. W. Thomson Walker, these speakers dealing respectively with organic causes, functional causes, and the thymus gland and status lymphaticus. The surgical aspect was dealt with by Mr. A. H. Tubby, and Dr. J. Blumfeld read a paper on deaths in children during anæsthesia. As might be expected, under the heading of medical causes there was a great deal of ground to be covered, and many graphic and startling cases were described. Dr. Macalister, under the subtitle of toxic and septic causes, mentioned a number of instances which had occurred in some industrial schools in Liverpool, where boys apparently in good health had suddenly died. In one such case there was an entertainment in progress which one of the boys had to leave because he was incessantly coughing, the cough being of an irritative nature. He was presented with an apple and an orange to comfort him for his disappointment, and then went to bed. Shortly afterwards he was found dead in bed with the apple and orange uneaten. But little was found post-mortem to account for the sudden death, which was probably due to pneumococcic toxæmia. Improvement in certain sanitary arrangements had been followed by a disappearance of such cases from the industrial schools. Sudden or rapidly ensuing death as a result of acute infectious disorders was referred to by several members of the Society, as also was sudden death from internal hæmorrhages in the early stages of purpuric affections, sudden death in cases of abscess and of tumour of the brain, and many other causes. Dr. Guthrie alluded to sudden death following the rapid healing of cutaneous eruptions. He was under the impression that the abuse of mercury might account for some instances. He now limited the dose of Hyd. c. cretâ to $\frac{1}{4}$ grain in children. But a case had occurred where no mercury had been given. In opening the difficult subject of functional causes, Dr. Parkinson remarked that all causes of death are really organic, though it is customary to classify as functional those which cannot be appreciated by clinical or post-mortem examination. Such cases include many deaths during convulsions in infants, in whom no gross lesion can be found after death. Instances are not unknown where death appears to be brought about by nervous excitement, and Dr. Parkinson mentioned the case of a boy who fell dead while watching a fight between two gipsies.

Much interest was aroused by the subject of enlarged thymus gland and the status lymphaticus, but, as was apparent from the remarks made by several members, more numerous and accurate pathological data are yet required before the condition can be properly understood.

Dr. Blumfeld classified deaths during anæsthesia into three divisions, according as death ensues previous to, during, or after operation, and mentioned some statistics obtained from the Registrar-General's reports which showed an astonishing number of deaths during anæsthesia in children under five years of age. He said it was a mistake to assume that chloroform was the only anæsthetic for infants, and when given to patients of tender age great care was necessary in the administration. Of the deaths occurring in the preliminary stages of anæsthesia—that is to say, before the commencement of operation, all or nearly all have followed the use of chloroform. It is important, as he pointed out, in giving chloroform to a child to gain its confidence and avoid frightening it as far as possible.

We should suggest that the Society for Study of Disease in Children would do well to give an early opportunity for a further discussion of sudden and unexpected death in children, as the subject has not only great academic interest but it has many practical bearings. The latter are illustrated by an example quoted by Dr. Cautley in which a nine months' old baby, illegitimate, insured and bottle-fed—three circumstances not conducive to longevity—suddenly dies from no obvious cause. It is clearly unsatisfactory that medical science cannot explain the death after a post-mortem examination has been made, but until more facts have been carefully collected we are likely to remain in this state of darkness.

